# An Electronic Phenotype Series for Measuring Adherence to the Wake Up and Breathe Protocol in Mechanically Ventilated ICU Patients

**DOI:** 10.21203/rs.3.rs-9954442/v1

**Published:** 2026-06-29

**Authors:** Andrew J King, Billie S Davis, Kathryn A Connell, Leigh A Bukowski, Kanupriya Soni, Susan Graf, Jeremy M Kahn

**Affiliations:** University of Pennsylvania; University of Pittsburgh; University of Pennsylvania; University of Pittsburgh; Beth Israel Deaconess Medical Center; University of Pittsburgh; University of Pittsburgh

**Keywords:** Mechanical ventilation, Electronic phenotype, Computable clinical guidelines, Spontaneous awakening trial, Spontaneous breathing trial

## Abstract

**Objective.:**

The Wake Up and Breathe protocol integrates daily spontaneous awakening and breathing trials to facilitate earlier weaning from mechanical ventilation, improving outcomes for patients with acute respiratory failure. Measuring adherence to this multistep, interprofessional practice is essential but is challenged by informatics complexity and the risk of metric-specific gaming. Our objective was to define and validate an electronic phenotype series enabling retrospective, scalable, and reliable measurement of protocol adherence using real-world electronic health record data from a multi-hospital warehouse, with the patient-day as the unit of analysis and agreement against manual chart review of 200 stratified patient-days assessed using Cohen's Kappa.

**Results.:**

Seven phenotypes were defined: two for upstream eligibility, four for protocol process steps, and one for a downstream outcome. Agreement with chart review ranged from moderate to near-perfect, with lower agreement linked to documentation granularity and rapid clinical events. This phenotype series offers a reusable framework for measuring adoption of the Wake Up and Breathe protocol and supports scalable quality measurement and retrospective analysis across ICU settings, though some protocol components remain unmeasured given current data availability.

## Introduction

Evidence-based protocols translate complex clinical guidelines into actionable bedside strategies. One example is the Wake Up and Breathe protocol for intensive care units (ICUs) [1], which integrates coordinated spontaneous awakening trials (SATs) and spontaneous breathing trials (SBTs) for patients receiving invasive mechanical ventilation for acute respiratory failure [2]. The protocol facilitates earlier ventilator weaning, a practice associated with lower mortality and shorter ventilation duration [3]. Ongoing measurement of protocol use is therefore essential, but two barriers hinder it.

First, performance measurement can inadvertently alter documentation. In a multihospital study of a financial incentive for SBT completion [4], hospitals with lower baseline adherence showed increased SBT rates, but the improvement was largely driven by increased documentation of eligibility exclusions rather than consistent gains in patient outcomes—illustrating the risk of gaming when metrics rest on documentation rather than objective data. Second, the protocol poses informatics challenges. As a recurring, multistep process, it depends on sequenced actions distributed across bedside monitors, clinical orders, and physiologic assessments, and coordinated among interprofessional team members. Progress is often response-dependent and partly non-deterministic, the protocol does not consistently incorporate upstream exclusions such as scheduled procedures, and fragmented documentation further complicates measurement.

To address these challenges, we define and validate an electronic phenotype series for measuring adherence to the Wake Up and Breathe protocol. Electronic phenotypes are algorithmically defined representations of clinical concepts derived from electronic health record (EHR) data [5, 6]. To capture protocol complexity, our definitions are both multifactorial—drawing on heterogeneous data elements—and sequential, such that the criteria for one phenotype depend on satisfying a preceding one. This enables more accurate, reproducible, and scalable measurement of adherence across health systems.

## Main text

### Phenotype definition

We first collated relevant clinical and implementation resources, with the most informative guidance from the Agency for Healthcare Research and Quality (AHRQ) [7], then organized the extracted criteria into structured, sequential definitions reflecting the protocol's multistep nature.

We also identified clinically relevant criteria that, while outside the formal protocol bounds, provide context for protocol applicability. For example, weaning is inappropriate if a patient is scheduled for an operating room procedure later that day. Operational decisions were made during this step. Because the protocol is typically executed once per 24-hour period, the patient-day was selected as the unit of analysis. Because the intent was retrospective quality measurement rather than real-time decision support, definitions permitted data that may lag the actual time of clinical decision-making, for instance assessing whether surgery occurred later in the day. To meet local quality goals, patients ventilated via a tracheostomy tube were excluded.

We then populated value sets using the institution's research data warehouse, which aggregates ICU data from 12 hospitals within a large integrated health system in the mid-Atlantic United States. Each criterion was assessed for digital availability—whether the warehouse could reliably determine if it was met. Criteria that were unavailable or unascertainable were excluded.

### Phenotype validation

We conducted a manual chart review on a stratified sample of 200 patient-days drawn from patients receiving invasive mechanical ventilation and judged potentially eligible for weaning. Cases were drawn from a range of ICUs to ensure variation across settings and patient characteristics, with sampling stratified to represent the value ranges of each definition component. Each case was independently reviewed by two trained abstractors, with disagreements resolved by a third reviewer. Agreement between phenotype and chart-review determinations was assessed using Cohen's Kappa (κ). The study protocol was approved by the University of Pittsburgh's Human Research Protection Office with a waiver of informed consent.

### Phenotype definitions

The final series comprised seven phenotypes, each with two to ten components representing distinct clinical criteria. Semi-structured definitions appear in Table 1. Each component is defined affirmatively—the phenotype is present only when all components are satisfied. Temporal logic used local time stamps relative to each calendar day. Many components reference standardized value sets, detailed in Table 2.

Two phenotypes (@ReceivingIMV and @WeanEligible) serve as eligibility criteria upstream of the protocol, defining the population to whom it should apply. Four (@SATEligible, @SATReceipt, @SBTEligible, @SBTReceipt) are process measures corresponding to the protocol's core steps. One (@SustainedExtubation) is a downstream outcome measure, chosen as more informative than the protocol's final “consider extubation” step.

Due to EHR documentation limitations, several original protocol components could not be included. For @SATEligible, the excluded components were high-frequency oscillatory ventilation, active seizures, alcohol withdrawal, agitation, active myocardial ischemia, persistent anxiety, persistent pain, respiratory distress, acute cardiac dysrhythmia, and diaphoresis. For @SBTEligible, agitation, inspiratory efforts, and active myocardial infarction were excluded. Outcome measures for SAT and SBT failure, and downstream process measures (e.g., sedation reinitiation or return to full ventilatory support), were also excluded due to insufficiently granular data.

### Validation

Validation results appear in Table 3. Cohen's Kappa ranged from moderate agreement for @SATReceipt to almost perfect agreement for @SATEligible and @SBTEligible [8]. Agreement was generally higher for eligibility phenotypes than for process phenotypes.

The lower agreement for @SATReceipt and @SBTReceipt was primarily attributable to rapid trial failures. If sedation was stopped and restarted within the same hour, our implementation of the @SATReceipt.NotReceivingContSedativeCheck component failed to register the SAT, as the data captured intravenous administration only at hourly intervals and rate changes—except at discontinuation. Similarly, when patients failed SBTs quickly, respiratory therapists often did not document a switch to an SBT ventilator mode, producing false negatives.

## Discussion

To support reliable process and outcome measurement of the Wake Up and Breathe protocol, we developed and validated an electronic phenotype series representing key stages of the coordinated awakening and breathing trial process. This work demonstrates both the feasibility of translating a complex, interprofessional ICU protocol with multiple interdependencies into structured digital definitions and the challenges of doing so with real-world EHR data.

Several lessons emerged. First, we deliberately decoupled phenotypes to prevent error propagation: we did not make @SBTEligible contingent on SAT success, both because SAT success was not calculated and because @SATReceipt showed only moderate agreement; decoupling prevented inaccuracies in the SAT phenotype from propagating into SBT analyses. Likewise, @SustainedExtubation was defined independently of upstream phenotypes to avoid excluding patients extubated despite not meeting prior criteria. Second, measuring partial progression through the weaning pathway—even without complete adherence—offers insight into systematic failures; patterns in missed SATs or SBTs may reflect overuse of neuromuscular blockade or coordination breakdowns, providing a basis for quality improvement. Third, the definitions rely on retrospective data including forward-looking indicators; near real-time decision support would require restructuring this logic to incorporate scheduling systems or manual entry. Fourth, identifying SATs from intravenous sedation data proved difficult because cessation of sedation is often not recorded. Finally, we included both continuous and intermittent intravenous sedatives to maximize sensitivity, which may slightly overestimate sedative exposure and may warrant revisiting based on local practices.

To support reuse, users should confirm that their use case aligns with the strengths of these definitions, which suit retrospective process and outcome measurement rather than real-time decision support. Local adaptation of value sets is essential, with attention to omitted concepts that may affect fidelity, and local validation is strongly recommended because documentation patterns vary across institutions and over time. While these definitions support pre–post evaluations, their greatest utility lies in enabling continuous monitoring to detect shifts in adherence, documentation behavior, or emerging clinical trends.

### Limitations

First, the series does not capture the full breadth of the protocol; it does not measure the success of awakening or breathing trials, nor clinical actions taken after failed trials. These were excluded mainly due to inconsistent availability of critical data elements—such as documentation of agitation, respiratory distress, or cardiac arrhythmia—needed to determine trial outcomes. These omissions limit completeness but do not undermine the series' value for measuring key processes and outcomes. Second, validation used a purposive rather than random sample; although only 200 patient-days were reviewed, selection captured a diverse range of cases, each independently reviewed by multiple reviewers, strengthening credibility. Finally, generalizability to other health systems may be constrained, as the definitions and value sets were developed within a specific data infrastructure and clinical environment; institutions should evaluate local documentation practices, coding standards, and workflow variations before adopting this approach.

## Figures and Tables

**Table 1.** Semi-structured phenotype definitions for retrospectively measuring weaning progression of patients ventilated via an endotracheal tube. A phenotype evaluates to True when all of its components evaluate to True. Relevant value sets are indicated with {{double curly braces}} and are available in Table 2.

Phenotype Name (Phenotype Identifier)	Component Description (Component Identifier)	Criteria — all times are relative to an individual calendar day
Receiving invasive mechanical ventilation (@ReceivingIMV)	Tube status indicates invasive mechanical ventilation (.TubeStatusCheck)	At 07:00 LT (local time), the most recent value of the flowchart variable tube_status was in the value set {{intubated_tube_status}}.
	Ventilator mode indicates invasive mechanical ventilation (.VentModeCheck)	At 07:00 LT, the most recent value of the flowchart variable ventilator_mode was in the value set {{invasive_mechanical_ventilation_mode}}.
Wean eligible (@WeanEligible)	Receiving invasive mechanical ventilation (.ParentPhenoCheck)	Meets @ReceivingIMV definition.
	The intubation procedure occurred prior to today (.NotNewIntubationCheck)	Between 15:00 LT the prior day and 07:00 LT, all values of the flowchart variable tube_status were in the value set {{intubated_tube_status}}.
	The intubation is not via a tracheostomy tube (.NotTrachedCheck)	Between hospital admission and 03:00 LT the next day, the value of the flowchart variable tube_status is never in the value set {{tracheostomy_tube_status}}.
	The ICU admission occurred prior to today (.NotNewICUAdmitCheck)	Between 15:00 LT the prior day and 07:00 LT, all values of the location history variable unit_name were in the value set {{icu_unit}}.
	The treatment is not limited to comfort measures only (.NotCMOCheck)	At 07:00 LT, the most recent value of the order variable code_status was in the value set {{CMO_code_status}}.
	No operating room visit today (.NoScheduledSurgeryCheck)	Between 07:00 LT and 07:00 LT the following day, none of the values of the location history variable unit_name were in the value set {{operating_room_unit}}.
SAT eligible (@SATEligible)	Receiving a continuous sedative (.ParentPhenoCheck)	Meets @WeanEligible definition.
	An intravenous sedative medication is being administered (.ReceivingContSedativeCheck)	Between 03:00 LT and 07:00 LT, at least one of the values of the medication administration variable medication_name is in the value set {{intravenous_sedative}}.
	A paralytic medication is not being administered (.NotReceivingParalyticCheck)	Between 03:00 LT and 07:00 LT, none of the values of the medication administration variable medication_name is in the value set {{neuromuscular_blockade}}.
	Oxygen saturation is above threshold (.OxygenSaturationCheck)	At 07:00 LT, the most recent value of the flowchart variable SpO2 was greater than 88%.
	Intracranial pressure (ICP) is within recommended limits (.ICPCheck)	At 07:00 LT, the most recent value of the flowchart variable ICP was less than 20 mmHg.
	Respiratory rate is below threshold (.RespiratoryRateCheck)	At 07:00 LT, the most recent value of the flowchart variable Respiratory Rate was less than 35/min.
SAT receipt (@SATReceipt)	SAT eligible (.ParentPhenoCheck)	Meets @SATEligible phenotype.
	No intravenous sedative medication is being administered (.NotReceivingContSedativeCheck)	At any period between 07:00 LT and 11:00 LT, none of the values of the medication administration variable medication_name is in the value set {{intravenous_sedative}}.
SBT eligible (@SBTEligible)	Eligible for weaning (.ParentPhenoCheck)	Meets @WeanEligible definition.
	Oxygen saturation is above threshold (.OxygenSaturationCheck)	At 07:00 LT, the most recent value of the flowchart variable SpO2 was greater than 88%.
	Fraction of inspired oxygen (FiO2) setting is within recommended limits (.FiO2Check)	At 07:00 LT, the most recent value of the flowchart variable FiO2 was less than or equal to 50%.
	Positive end-expiratory pressure (PEEP) setting is within recommended limits (.PEEPCheck)	At 07:00 LT, the most recent value of the flowchart variable PEEP was less than or equal to 7.5 cm H2O.
	Respiratory rate is below threshold (.RespiratoryRateCheck)	At 07:00 LT, the most recent value of the flowchart variable Respiratory Rate was less than 35/min.
	A paralytic medication is not being administered (.NotReceivingParalyticCheck)	Between 03:00 LT and 07:00 LT, none of the values of the medication administration variable medication_name is in the value set {{neuromuscular_blockade}}.
	Intracranial pressure (ICP) is within recommended limits (.ICPCheck)	At 07:00 LT, the most recent value of the flowchart variable ICP was less than 20 mmHg.
	Vasopressor administration below contraindication levels (.VasoStrictCheck)	Between 03:00 LT and 07:00 LT, none of the values of the medication administration variable medication_name is in the value set {{vasopressor_strict_exclusion}}.
	Vasopressor administration below contraindication levels (.VasoDoseTwoCheck)	Between 03:00 LT and 07:00 LT, when the value of the medication administration variable medication_name is in {{vasopressor_twomcg_exclusion}}, none of the corresponding values for medication_rate are greater than 2 mcg/kg/min.
	Vasopressor administration below contraindication levels (.VasoDoseFiveCheck)	Between 03:00 LT and 07:00 LT, when the value of the medication administration variable medication_name is in {{vasopressor_fivemcg_exclusion}}, none of the corresponding values for medication_rate are greater than 5 mcg/kg/min.
SBT receipt (@SBTReceipt)	SBT eligible (.ParentPhenoCheck)	Meets @SBTEligible definition.
	Ventilator mode indicates SBT (.SBTVentModeCheck)	Between 07:00 LT and 11:00 LT, at least one value of the flowchart variable ventilator_mode was in the value set {{sbt_mode}}.
Sustained extubation (@SustainedExtubation)	Receiving invasive mechanical ventilation (.ParentPhenoCheck)	Meets @ReceivingIMV definition.
	Patient received an extubation procedure (.ExtubatedCheck)	Between 07:00 LT and 07:00 LT the following day, at least one value of the flowchart variable tube_status is in the value set {{extubated_tube_status}} OR at least one value of the flowchart variable ventilator_mode is in the value set {{extubated_ventilation_mode}}.
	Tube status does not indicate invasive mechanical ventilation (.NotReintubatedCheck)	Between 07:00 LT the following day and 07:00 LT two days later, none of the values of the flowchart variable tube_status are in the union of the value sets {{intubated_tube_status}} and {{tracheostomy_tube_status}}.
	Ventilator mode does not indicate invasive mechanical ventilation (.NotInvasiveModeCheck)	Between 07:00 LT the following day and 07:00 LT two days later, none of the values of the flowchart variable ventilator_mode are in the value set {{invasive_mechanical_ventilation_mode}}.
	Patient did not die within 48 hours (.Alive48hrsCheck)	Between 07:00 LT and 07:00 LT two days later, patient death is not documented.

**Table 2.** Value set definitions.

Value Set Identifier	Domain	Variable	Value Set
{{intravenous_sedative}}	Medication administration	medication_name	Fentanyl; Hydromorphone; Morphine; Ketamine; Dexmedetomidine; Lorazepam; Midazolam; Propofol
{{neuromuscular_blockade}}	Medication administration	medication_name	Cisatracurium; Rocuronium; Succinylcholine; Vecuronium
{{vasopressor_twomcg_exclusion}}	Medication administration	medication_name	Norepinephrine
{{vasopressor_fivemcg_exclusion}}	Medication administration	medication_name	Dobutamine; Dopamine
{{vasopressor_strict_exclusion}}	Medication administration	medication_name	Isoproterenol; Epinephrine; Phenylephrine; Vasopressin
{{invasive_mechanical_ventilation_mode}}	Flowsheet	vent_mode	AC; APRV; BiLevel; HFOV; PAV; PCV; PRVC; SIMV
{{extubated_ventilation_mode}}	Flowsheet	vent_mode	NC; NIV; Other off vent
{{sbt_mode}}	Flowsheet	vent_mode	CPAP; PSV; T-piece^a^
{{intubated_tube_status}}	Flowsheet	tube_status	Intubated; Re-intubated
{{extubated_tube_status}}	Flowsheet	tube_status	Extubated; Self-Extubated
{{tracheostomy_tube_status}}	Flowsheet	tube_status	New Tracheostomy; Pre-Existing Trach
{{icu_unit}}	Location history	unit_name	Not provided (hospital-specific)
{{operating_room_unit}}	Location history	unit_name	Not provided (hospital-specific)
{{CMO_code_status}}	Order	code_status	Comfort Measures Only

a Not used locally but common in many health systems.

**Table 3.** Contingency table for the manual chart review of an informative set of 200 patient-days meeting the phenotype determination for @ReceivingIMV. Columns report the number of patient-days judged Yes or No on manual chart review.

Electronic Phenotype	Phenotype Determination	Chart Review: Yes	Chart Review: No	Kappa (95% CI)
@ReceivingIMV	Yes	197	3	N/A
	No	N/A	N/A	
@WeanEligible	Yes	120	2	0.68 (0.57, 0.78)
	No	26	49	
@SATEligible	Yes	95	6	0.82 (0.73, 0.92)
	No	5	40	
@SATReceipt	Yes	31	21	0.53 (0.38, 0.67)
	No	9	85	
@SBTEligible	Yes	91	5	0.83 (0.74, 0.93)
	No	6	44	
@SBTReceipt	Yes	56	2	0.68 (0.56, 0.79)
	No	22	66	
@SustainedExtubation	Yes	15	5	0.61 (0.43, 0.78)
	No	11	166	

## Data Availability

The datasets generated and/or analysed during the current study are not publicly available due to institutional policies protecting the privacy of individuals whose data were used, but are available from the corresponding author on reasonable request.

## Abbreviations

AHRQ: Agency for Healthcare Research and Quality
EHR: electronic health record
ICU: intensive care unit
SAT: spontaneous awakening trial
SBT: spontaneous breathing trial
